# Proposal of Combined Noise and Hand-Arm Vibration Index for Occupational Exposure: Application to a Study Case in the Olive Sector

**DOI:** 10.3390/ijerph192114345

**Published:** 2022-11-02

**Authors:** Raquel Nieto-Álvarez, María L. de la Hoz-Torres, Antonio J. Aguilar, María Dolores Martínez-Aires, Diego P. Ruiz

**Affiliations:** 1 Department of Architectural Graphic Expression and Engineering, University of Granada, Av. Severo Ochoa s/n, 18071 Granada, Spain; 2 Department of Building Construction, University of Granada, Av. Severo Ochoa s/n, 18071 Granada, Spain; 3 Department of Applied Physics, University of Granada, Av. Severo Ochoa s/n, 18071 Granada, Spain

**Keywords:** physical agents, noise, vibration, HAV, workers, olive sector

## Abstract

In many production and industrial sectors, workers are exposed to noise and hand-arm vibrations (HAV). European directives have established the maximum limit values or exposure action values for noise and vibration independently. However, in many cases, workers who endure hand-arm vibration also receive high noise levels. This research suggests a procedure to aid the establishment of precautionary measures for workers with simultaneous exposure to both physical agents. This procedure defines a combined index based on the energy doses for both noise and HAV. From this combined index, the suggested methodology allows a recommended exposure time for workers with simultaneous noise and HAV exposure to be calculated. This methodology can be adapted to tackle the relative importance assigned to both agents according to the safety manager and new knowledge on combined health effects. To test this method, a measurement campaign under real working conditions was conducted with workers from the olive fruit-harvesting sector, where a variety of hand-held machinery is used. The results of the study case show that the suggested procedure can obtain reliable exposure time recommendations for simultaneous noise and HAV exposures and is therefore a useful tool for establishing prevention measures.

## 1. Introduction

Scientists, experts and numerous official bodies such as the World Health Organisation (WHO) or the European Economic Community (EEC) have recognised noise and vibration as a health hazard and their effects have been regarded as an increasingly important health problem [1,2]. In terms of noise, for example, health effects of environmental noise exposure (i.e., noise in the living environment such as road traffic, railway, aircraft, building and construction noises or industrial noise) have been extensively studied in scientific literature. These effects range from purely physiological disorders, such as progressive hearing loss, to psychological disorders, such as irritation and fatigue, leading to dysfunctions in daily life [3,4,5].

In addition, other research has focused on health effects of occupational noise exposure (in the working environment) specifically. Indeed, workers are exposed in their workplace to various environmental factors that can affect their health. These factors include physical agents such as vibration [6,7,8] and noise [9,10,11], which can cause various pathologies and adverse effects. Nowadays, the exposure of workers to both physical agents has increased as all productive sectors have evolved towards mechanisation, and so the probability of suffering from these adverse health effects has increased. For example, the increased use of hand tools (electric, combustion, pneumatic and hydraulic) causes exposure to noise and HAV. For this reason, their effects have been analysed in many productive sectors such as construction [12,13], agriculture [14,15,16], forestry [17,18], metallurgy [19], textile industry [20], chemical manufacturing [21], automobile manufacturing [22], mining [23], the mining industry and mechanical engineering [24] etc. The issue of occupational noise and vibration exposure is so relevant that the Sixth European working conditions survey [25] reports that 28% of all workers are exposed to high noise levels for more than a quarter of the working day in different labour sectors. Furthermore, 20% of workers are exposed to vibrations produced by tools or machines for more than a quarter of the working time. These data show the relevance and importance of the problem in the occupational risk assessments.

Despite advances in preventive and protective measures to limit such effects, they are still present today in a large number of occupational activities. This research focuses on the establishment of preventive measures in workers with simultaneous exposure to both physical agents as a reinforcement of safety.

In terms of health effects, on the one hand, it is well established that exposure to occupational noise also causes health effects such as vascular problems [26], heart disease and cardiovascular disease [27,28,29], insomnia [30], achievement and memory problems [31]. In this matter, a causal relationship between noise exposure and hearing loss has been clearly demonstrated [11,32,33,34,35]. One of the most usual consequences is tinnitus [36,37]. Boger et al. [38] indicated the prevalence of tinnitus of workers exposed to occupational noise could be as high as 66%. Another problem commonly reported is the extended high-frequency hearing loss and poor speech perception [34].

On the other hand, exposure to vibrations can cause other health effects. One of the most studied and best-known effects with regard to exposure to hand-arm vibration (HAV) is Raynaud’s syndrome [39,40,41], also known as white fingers syndrome. Raynaud’s syndrome is an ischemic response in the skin of the fingers, which reduces blood flow and causes discoloration [42]. Burström et al. [43] also demonstrated that cold environment also increases the risk of white fingers in workers occupationally exposed. In addition to this effect, HAV may cause other health effects; among the most commonly reported are musculoskeletal disorders [44,45], vascular problems [46] and neuro-sensory problems with loss of manual dexterity [47].

### 1.1. Simultaneous Exposure to Noise and Vibration

Numerous studies have also reported research on the combined effects of noise and other factors. Golmohammadi, R. and Darvishi, E. [9] presented a thorough review of effects of combined exposure to noise and other exposures, such as chemical agents and physical factors (i.e., lighting, heat, etc.). In their review they also paid attention to the state-of-the-art insights in differences in effects of noise exposure by personal characteristics such as age, gender, genetic background, etc. From this research, an increased incidence of health effects in workers exposed to simultaneous vibration and noise has been reported. This increased incidence has been described from several aspects.

On the one hand, relevant and early attention was paid to study the possible association between Raynaud’s syndrome and hearing loss, i.e., Iki et al. (1987) [48] and Miyakita et al. in (1990) [49] found a decrease in finger temperature when hearing protectors are not used at the same level of vibration. In this line of research, Pyykkö et al. [50] studied the shipyard workers, forestry workers, metal workers and patients referred to a clinic. They reported that workers with vibration-induced Raynaud’s syndrome were more susceptible to hearing loss at 4 kHz than workers exposed to noise alone. In the same regard, Turcot et al. [18] concluded that combined exposure to noise and vibration should be considered as possible risk factors for developing hearing loss.

Other studies in the same research topic led to similar conclusions. Duan D. P. et al. [22,51] detected the increased risk of adverse effects on hearing function when the worker is additionally exposed to vibration under the same noise conditions in the workspace. In the same sense, other authors such as Pettersson et. al. [52,53,54,55] detected a higher probability of hearing problems in workers who already suffered Raynaud’s syndrome. In terms of the influence of noise in probability of appearance of the Raynaud’s syndrome, other studies by Stjernbrandt et al. [56] found a higher probability of developing Raynaud’s syndrome among individuals exposed to occupational noise than only under the effects of HAV exposure. Turcot [18] confirms previous findings of greater hearing loss at higher frequencies in workers with Raynaud’s syndrome.

In terms of the association of Raynaud’s syndrome and hearing loss, current research shows that the appearance of this association is highly likely when both physical agents are involved, but more research is needed to quantify these effects.

On the other hand, a large number of studies in this subject have also been focused on the effect on workers’ comfort or psychological effects [57]. It has been suggested that the psychological and stressful problems created by both noise and whole-body vibration may lead to accidents in professional drivers [58] and affect the ability to work, leading to reduced physical and mental performance [59]. Other researchers such as Huang and Griffin [60] showed that the sensation of discomfort increases when there is simultaneous exposure to noise and whole-body vibration. Another study by Kim et al. [61] detected increased headaches and visual fatigue under the combined effect of noise and vibration in workers. Psychological effects have also been found in workers subjected to HAV and noise simultaneously, such as psychological anxiety and neurological problems [62], nervous system related disorders [63], mental health problems such as depression [57] and insomnia problems [64], even at levels under the recommended limits by the standards, and they may also lead to other disturbances which, although they might seem less important, cause discomfort to the operator, such as an increased sweating of the hands when handling hand tools in the presence of noise [65]. Nari et al. [64] revealed an association between occupational noise and vibration exposure and insomnia, both individually and simultaneously.

In terms of comfort and psychological effects, current research shows that increased discomfort is highly likely when both physical agents are involved, but more research is also needed to quantify these effects and additional studies and research are required to further understand this relationship.

Accordingly, since the appearance of health effects from combined exposure to both noise and vibration is highly likely, some interesting research has looked into the assessment of the possible association between them, assessing both the possible increase in health effects on workers and the appearance of new effects. If the literature review focuses on the effects of combined exposure to noise and specifically hand-arm vibration, the first research study on the effects of combined exposure was the research of Petterson et al. [55]. They suggested that the combined exposure effects could be due to the additional noise of the vibrating tool. Finally, they underlined that these results cannot be generalized to elderly and unhealthy subjects exposed at the workplace because the sample they analysed was constituted of healthy young subjects with no history of occupational exposure. A further insight in the published literature into the possible relation or quantification of this effect in simultaneous exposure to noise and vibration in general is found in several interesting research studies. As illustrative examples of the findings in this subject, the research by Sisto R. et al. [66] and Ljungberg and Neely [59] is remarkable. Sisto R. et al. [66] conducted an experimental study with 12 volunteers. They concluded that a synergistic effect of noise and vibration in inducing cochlear damage seems to be present and it is possible to make quantitative estimates of this effect. This idea contradicts those of Ljungberg and Neely [59] who claim neither synergistic nor antagonistic effects were observed from the combined noise and vibration exposure. Other work carried out by Zhu et al. 1997 [67] concluded that noise exposure is essential to produce an increase in temporary threshold shift of hearing and, together with noise exposure, hand-arm vibration can enhance the effect of noise on hearing by producing a higher sympathetic activity which might cause greater vasoconstriction in the inner ear, but no clear quantification on exposure limits was reported.

A more in-depth review about the combined effects of occupational exposure to noise and other risk factors carried out by Golmohammadi and Darvishi [9] concluded that the current research evidence shows that there is high evidence that the exposure to vibration exacerbates all noise effects. Nevertheless, the possible various interactions between noise and HAV should be studied further. In the same sense, Sisto et al. [66] concluded that there is a risk that exposure to mild noise levels could interfere with the exposure to vibration, enhancing the adverse effect on the hearing function. This should be considered to optimise prevention strategies at the workplace.

### 1.2. Standards and Regulations

International standards define two exposure values for these physical agents. On the one hand, Exposure Action Value (EAV) is a daily amount of vibration or noise exposure above which employers are required to take action to control exposure. The Exposure Limit Value (ELV) is the maximum amount of vibration or noise an employee can be exposed to on any single day. In this regard, the EU Directive [68] establishes the limit values for vibration; the Exposure Limit Values (ELV) are set up in 5 m/s^2^ and Exposure Action Value (EAV) in 2.5 m/s^2^. The same values are recommended by The American Conference of Governmental Industrial Hygienists (ACGIH) [69], in its latest publication of TLVs and BEIs [70]. Unlike the previous ones, NIOSH [71] does not provide a limit value but it establishes recommendations to protect the health of workers. In terms of measurement procedure, international standards also establish the HAV vibration measurement procedure; in this case both ISO 5349-1:2001 and ISO 5349-2:2002 [72,73] as well as ANSI S2.70 [74].

In noise regulations there are more differences in ELV, with values ranging from 85 to 90 dBA. Thus, the European Directive [75] establishes 87 dBA as (ELV) value while USA-OSHA raises it up to 90 dBA. The strictest recommendations are established by NIOSH and ACGIH in TLVs and BEIs [70], since they both establish the ELV equal to 85 dBA. This is the same value that the Directive-2003/10/CE [75] establishes for the EAV as well as ISO 1999:2013 [68], so these values for noise are widely applied to perform exposure assessments. Since these EAVs in international standards are widely used, in this research they are adopted as a basis of providing a reference, but in case other occupational standards are used, the EAV should be substituted in the combined index proposal.

In Europe, manufacturers, suppliers and importers are affected by Directive 2006/42/CE [76] relative to machinery. It establishes that machinery must be designed and constructed in such a way that risks resulting from the emission of airborne noise are reduced to the lowest level. In the same way, the machinery must be designed and constructed in such a way that risks resulting from vibrations produced by the machinery are reduced to the lowest level. The values supplied by the manufacturers allow establishing protection measures at work but some studies such as [77] find some differences between the values provided by the manufacturer and those measured in real working conditions, which may cause the risk to be underestimated. This fact makes it necessary to carry out experimental risk assessments in order to design safe working procedures that minimise the exposure of workers to these risks. For this design, it is essential to establish recommendations or criteria based on time exposure, taking into account the combined exposure to both physical agents.

It should be noted that noise regulations established for occupational settings do not protect 100% of workers from NIHL, but rather strike a balance between hearing conservation and economic development (Shepard et al. [34]).

### 1.3. Main Hypothesis and Objectives of This Research

The main objective of this research is to reinforce the preventive measures in workers with simultaneous exposure to both noise and HAV in their occupational activities, i.e., as a reinforcement of safety in work. The main aim is to propose a combined index to suggest a maximum exposure time to both agents, thus providing a way to calculate this precautionary exposure time based on physical measurements, and so discouraging the use of rule-of-thumb criteria.

With this objective, this research aims to propose a combined index which accounts for the total energy received by the worker coming from both noise and HAV. The proposed index is based on the calculation of the combined energy dose calculated from the EAV prescribed by the standards. On this basis, a methodology is proposed to allow recommendations on exposure time to be adopted for minimizing the adverse health effects on workers.

Thus, the proposed index is based on the additive energy dose coming from both physical agents. The foundation of this proposal relies on the following main hypotheses 1 and 2:

**Hypothesis** **1.** *The current occupational noise and HAV regulations independently applied may underestimate the adverse health effects of combined exposure, so it is advisable to reinforce the preventive measures in workers with simultaneous exposition to both noise and HAV in their occupational activities.*

**Hypothesis** **2.** *The interaction between noise and HAV exposures should be considered as additive in terms of exposure to energy in developing the suggested procedure (i.e., the combined index). At the current state of the research, there is not an unambiguous relationship and quantification of this association between both physical agents, so a cautious approach is taken and a reasonable approximation to the total effect caused by a combination of noise and vibration can be determined from a summation of the effects of the individual agents.*

The rationale of these hypotheses was previously discussed in the preceding section. The first hypothesis comes from the health effects reported upon the scientific literature review where there are many signs and evidence of health effects of these agents in interaction. As a specific example to support this hypothesis, the work of Shepard et al. [34] concludes that the current occupational and environmental noise regulations may significantly underestimate the adverse health and societal cost of noise-induced hearing loss.

The second hypothesis is taken as a starting point for the combined index proposal. From the literature review on combined effects on noise and vibration, it can be concluded that the current research evidence shows that there is high probability that the exposure to vibration increases some noise effects, and vice versa, i.e., the noise exposure could interfere with the effects of exposure to vibration (Hypothesis 1). At the current state of the research, there is not a clear relationship and quantification of this association between both physical agents. At this stage of the research, it is advisable to optimize prevention strategies at the workplace until research on health effects of combined exposure can clearly establish the nature of the association between these agents. For the sake of implementing such a prevention strategy, a systematic literature review was performed on combined health effects in the preceding section. This review leads to the conclusion that the interaction between these two exposures is not well known at the current stage of research, and there is a lack of undeniable combined exposure models, i.e., there is no clear evidence that these effects should be considered as synergistic or not and how this could transfer to the exposure models.

In this context, the rationale of the proposed approach in this research lies in the current dose–response assessment methodology used previously in other risk assessment contexts, such as in the chemical agents’ case [78]. The conceptual framework supporting this proposal comes from the limited current knowledge on health effects coming from combined exposure. In this case a cautious approach is recommended to ensure that simultaneous exposure to noise and vibrations under their respective exposure limit values is properly addressed. In this case, an exposure index based on the sum of the energy exposures to each of the component divided by their respective exposure limits in terms of energy is a logical choice. These exposure limits are taken from the workers’ energy exposure based on the noise energy dose (Directive-2003/10/CE) [75] and HAV energy dose (ISO 5349-1:2001 and ISO 5349-2:2002) [72,73]. Thus, the energy dose concept and the criteria levels used in this research rely on the international standards. In summary, at the current stage of knowledge, this combined effect could be considered as additive, unless new information becomes available to indicate that the effects are synergistic, for example.

With the available information, this cautious approach is adopted, and the combined effect is considered additive in terms of exposure energy to develop the proposed method. 

As an example of the application of the proposed methodology, an experimental study is carried out in the olive sector, specifically in olive harvesting, analysing the exposure to both noise and HAV from a hook type olive harvester with combustion. To this aim, some measurement sensors and equipment were used to obtain data from noise and HAV, and some time exposure recommendations are given for this study case.

## 2. Occupational HAV and Noise in the Olive Oil Sector

One of the sectors in which mechanisation has been most evident throughout history has been agriculture. Among the sub-sectors, the olive oil sector has been no exception. 

Olive oil consumption has increased in all countries, even in Spain, where it was already traditionally consumed as the main dietary fat (Berbabeu et al. [79]). Spain is the world’s leading producer of olives, with a third of the total production according to data from the International Olive Council IOC [80]. The cultivated area of olive groves in Spain extends to 2.7 million hectares, of which Andalusia accounts for 60% according to data from the Survey on Surface Areas and Yields ESYRCE 2020 [81].

The sector has increased its productivity and competitiveness by incorporating machinery that reduces harvesting times. Deboli et al. [82] show that productivity can be tripled with the use of hand-guided electric machines. Machinery manufacturing companies offer a wide range of hand-guided tools (comb, hook, flap, beater, comb, hook, flap and beater). The most commonly used in Andalusia are hook and comb type harvesters.

This fact is very relevant from an economic point of view, as olive harvesting costs can account for up to 50% of the sale price of the product, according to Bernardi et al. [83]. Increased mechanisation can reduce these costs by 40–48% if handheld portable equipment is used, as demonstrated by Sperandio et al. 2017 [84].

Many workers are involved in these tasks. The number of working days in harvesting in an average production season in the region of Andalusia can amount to about 19 million, according to data from the “Olive grove production capacity 2021/2022” report in [85].

There are few studies on the impact of noise and HAV exposure in the olive sector. Some studies on hand-harvesters [86,87,88,89,90,91,92,93,94,95,96] only assess the HAV exposure received by the worker, with values almost always above the EAV set by Directive 2003/10/EC [75] and sometimes above the ELV.

Studies on the effects of both physical agents in olive harvesting work are scarce and give different results depending on the type of tool analysed and its power source. In comb-type harvesters with electric motor flap type portable harvesters, Cakmak et al. [97] found noise values lower than EAV (80 dBA) and vibration values above EAV (2.5 m/s^2^). Saracoglu et al. [98] evaluated hook type olive harvesters with combustion engine and obtained different vibration results for the right and left hand, but above EAV and higher noise values ELV (87 dBA).

In a previous study, the authors of the present article [99] presented the first results of the evaluation of the physical agents in olive harvesting, where the most commonly used hand-harvesters were evaluated. The noise values obtained were above EAV and ELV depending on the machine and the HAV levels were higher than ELV. In addition, all values were higher than those declared by the equipment manufacturer.

In general, the studies found on the machinery used during olive re-picking give varied results, exceeding the permitted values in most cases, but in any case, they are limited to measuring noise and vibration. Therefore, the high levels of exposure to noise and HAV in this sector justify their choice for the application of the proposed combined assessment index in the present study.

## 3. Materials and Methods

### 3.1. Data Acquisition and Testing Method

The testing method for determining the HAV and noise dose and the sampling time depends on the type of machinery being measured. Since the HAV and noise levels received by the worker are going to be assessed, different noise and vibration measurement sensors shall be placed on the worker themselves, as described below.

The level of vibration received by the operator has been carried out in compliance with the standard measurement and evaluation of human exposure to hand-transmitted vibration (Practical Guidance for Measurement at the Workplace. ISO 5349-1:2001 and ISO 5349-2:2002 [72,73]).

The measurement should last long enough to ensure reasonable statistical accuracy and for the vibration to be typical of the exposure being evaluated. The standards ISO 5349-1:2001 and ISO 5349-2:2002 [72,73] establish that the total duration of the vibration signal must be at least 1 min. For hand-guided tools and machines with all types of power sources (electric, hydraulic, pneumatic, internal combustion, etc.), ISO-20643:2008 [100] states that the measurements should be carried out in three series of five tests each, with a different experienced operator for each series.

For this reason, three series have been selected, each with one operator. The selected operators are experienced in the handling of the tool. In the work process, for each series, or, in other words, for each operator, five periods of at least 15” were taken, so that each sample lasted at least 75”, with a minimum of 1 min duration. 

The noise assessment was carried out according to ISO 1999:2013 [101]. It does not state the measurement time of the noise signal received by the worker, but it should be measured for a representative time of the dose. It has been measured in parallel during the same 15”, i.e., in simultaneous periods.

In order to measure the noise and vibration values received by the operator, the measurements were made when the hand tool was used during the actual working day, under normal conditions of use.

### 3.2. Equipment Used for Data Acquisition

**Figure 1:**
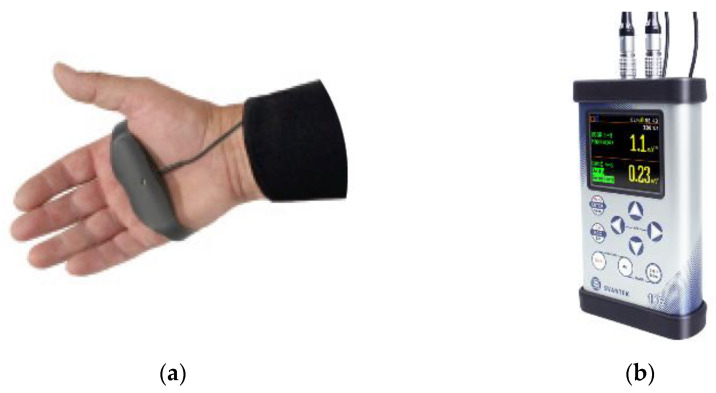
(**a**) Sensor placed on the worker’s hand; (**b**) SVAN 106 Four-Channel Sound and Vibration Analyser.

The equipment chosen to measure vibrations exposure was a human vibration meter (SVAN 106 equipment, SVANTEK) (Figure 1b). The vibrations transmitted to the hand–arm system was measured with a triaxial accelerometer (SV 105, Svantek). Accelerations were measured for both right and left hands. The accelerometer was placed on the handgrip (See Figure 1a) in the centre of the grip area of the machine handle, as this is where the most representative evaluation of the vibrations transmitted to the hand is obtained.

Vibration is measured in the three orthogonal axes according to ISO 5349-1:2001 and ISO 5349-2:2002 [65,66] standards. Subsequently, the Wh-weighting is established to analyse the signal and obtain values comparable with the maximum doses established in the standard. The values are studied in the frequency range covered by the octave bands from 8 Hz to 1000 Hz.

**Figure 2:**
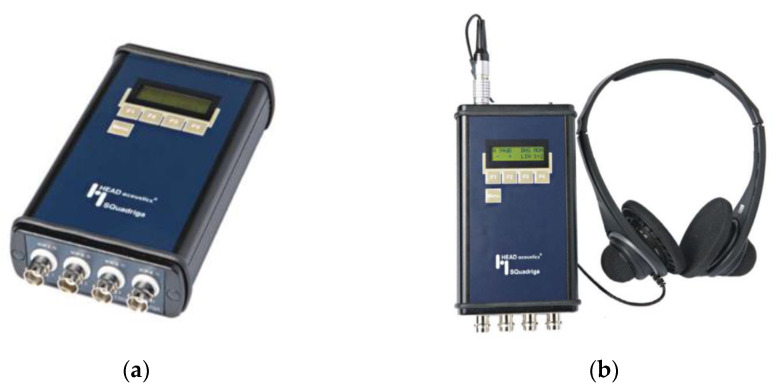
(**a**) SQuadriga Headphone Booster; (**b**) SQuadriga Headset BHS.

In the case of noise measurements, a SQuadriga I analyser was used to made binaural recordings at the worker position (Figure 2). The headset was placed over the worker’s ears. Noise level measurements were analysed with Artemis software.

### 3.3. Calculation of Exposure Level

The assessment of the exposure level to HAV is based on the calculation of daily exposure, which can be measured using the method of ISO 5349-1:2001 and ISO 5349-2:2002 [72,73]. 

**Figure 3:**
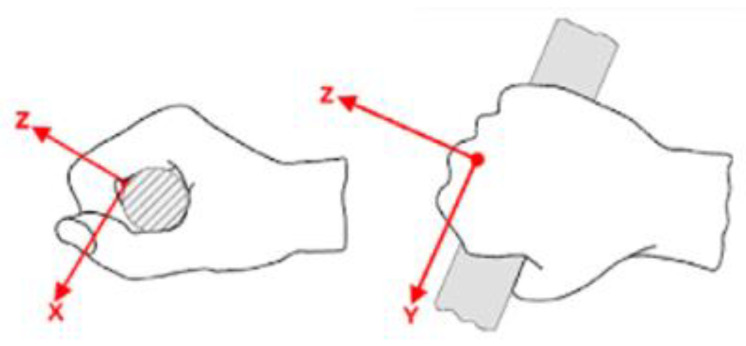
Axes direction defined in relation to the body hand (hand reference system).

The measured vibration magnitudes are expressed in Equation (1). The daily exposure *A*(8) is expressed as the equivalent continuous acceleration over an eight-hour period, calculated as an rms value.

(1) $$A_{i\left(8\right)}=a_{hv\;\;\;}\sqrt{\frac{T_{exp}}{T_{0}}}$$

where $a_{hv}$ is the magnitude of the vibration from the source producing it in m/s^2^; $T_{exp}$ is the duration of exposure to vibration $a_{hv}$ in seconds; $T_{0}$ is the 8-h reference period (28,800 s) and $a_{hv}$ is expressed as shown in Equation (2).

(2) $$a_{hv}=\sqrt{a_{hwx}^{2}+a_{hwy}^{2}+a_{hwz}^{2}}$$

where $a_{hwx},a_{hwy}$ and $a_{hwz}$ are the rms values of the acceleration of the vibrations to the hand, weighted in frequency after measuring the vibratory surface in contact with the hand on three orthogonal axes *x*, *y*, *z*, expressed in m/s^2^, see Figure 3. In the proposed scheme in this research, vibrations are considered in the three directions and measured accordingly to obtain the $a_{hv}$ value.

On the other hand, a magnitude frequently supplied by the manufacturer is the equivalent vibration, $a_{hv,eq}$_._ This value is calculated based on the acceleration values of vibration $a_{hv}$ for each handle, as well as the mode of machine operation Equation (3).

(3) $$a_{hv,eq}=\sqrt{\frac{1}{T}\overset{n}{\underset{i=1}{\sum }}a_{hwi}^{2}\;t_{i}}$$

The assessment of the exposure level to noise is based on the Directive-2003/10/CE [75], and measures noise exposure defined in the ISO-1999:2013 [101] standard.

The A-weighted equivalent continuous level $L_{Aeq,T}$ is obtained from Equation (4).

(4) $$L_{Aeq,T}=10log\left[\frac{\frac{1}{T}\int_{t1}^{t2}PA^{2}\left(t\right)dt}{P_{0}^{2}}\;\right]\;\mathrm{dB}\left(A\right)$$

where *PA* is the weighted sound pressure and $P_{0}$ is the reference pressure value (20 μPa), during a specified time interval of duration *T* ($t_{1}$ = 0 is the time at the beginning and $t_{2}\;$is the time at the end of the measurement).

To obtain the daily noise exposure level ($L_{Aeq,d}$) for an 8-h working day, the following equation is used (5):

(5) $$L_{Aeq,d}=L_{Aeq,T}+10log\left[\frac{T}{T_{0}}\;\right]\;\mathrm{dB}\left(A\right)$$

where ($L_{Aeq,T}$) is obtained from Equation (4), *T* is the actual time duration and $T_{0}$ is the reference time duration of the working day ($T_{0}$ = 8 h).

The daily noise exposure level ($L_{Aeq,d}$) is the parameter used as a risk predictor. The $L_{Aeq,d}$ is dependent on time and noise exposure levels during a nominal eight-hour working day. The ELV and the EAV with respect to the $L_{Aeq,d}$ are fixed in the Directive-2003/10/CE [75].

On the other hand, Directive 2002/44/EC [68] also establishes these limits for vibrations exposure. Both Directive 2002/44/CE and 2003/10/EC define the exposure limit values: the daily ELV and the daily EAV. Table 1 show the values defined in both directives.

The Directive 2006/42/CE [76] establishes that the manufacturer must declare the different emission values of the machine: the A-weighted emission sound pressure level at workstations, where this exceeds 70 dB(A), the peak C-weighted instantaneous sound pressure value at workstations, where this exceeds 63 Pa (130 dB in relation to 20 μPa), and the A-weighted sound power level emitted by the machinery, where the A-weighted emission sound pressure level at workstations exceeds 80 dB(A). It must also indicate under what conditions the test has been carried out. In the case of HAV, the manufacturer must indicate the vibration total value to which the hand-arm system is subjected, if it exceeds 2.5 m/s^2^. In the case of the type of hand-guided machinery that is going to be assessed, the measurement conditions are carried out according to ISO 11201:2010 standard [102] and for vibration ISO-20643:2008 standard [100]. However, these values are not always clearly declared and shown by the manufacturer, as shown by Nieto-Álvarez et al. [99]. This makes it difficult for safety managers to take adequate measures to protect workers. 

## 4. Results: Proposal of a Combined Index and Methodology for Its Calculation

The response of the human body to noise and vibration (health effects and annoyance) is related to the energy transmitted to the body by both the vibrations and noise. Based on this fact, much research has been made in developing dose–response relationships between the noise or vibration exposure and annoyance or health effects (for example in the recent books by Murphy E., 2014 [103] and Mansfield, N.J. 2004, [104] there is a comprehensive explanation on this topic). Therefore, since Directive-2003/10/CE [75] established the upper EAV for the daily (8 h) Equivalent Continuous Noise Pressure Level in dB(A)) and Directive 2002/44/EC [68] established the EAV for the daily (8 h) Acceleration Exposure *A*(8) (Table 1), it can be interesting to establish a criteria for the Combined Exposure Time ($T_{Cexp}$). This combined time will be established by considering both physical agents, since often there is a combined exposition in workers, such as in the olive sector.

**Figure 4:**
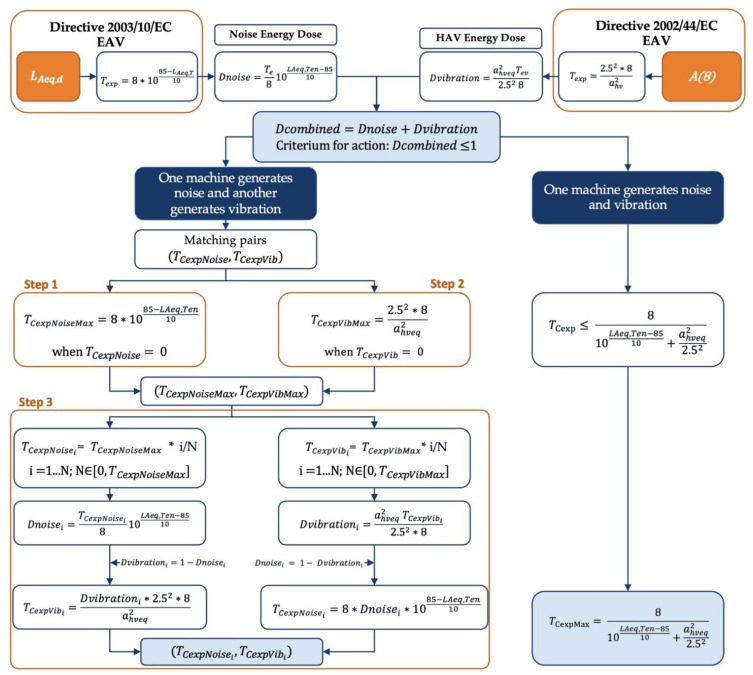
Flowchart of the proposed methodology.

For the sake of clarity, the following Figure 4 shows a flowchart of the proposed methodology for the main two cases of only one source of exposure (i.e., a machine generates both noise and HAV), and the case when the exposure to these physical agents originates from different noise and HAV sources.

From this figure, it can be followed that if one source generates both noise and vibration there will only be one maximum exposure time to meet the combined index below the EAV values. In case of two sources, the first step is to calculate the maximum time for noise on the one hand, and for vibration on the other hand, assuming that there is only one of these physical agents. The third step would be to establish the pairs of exposure times, depending on the doses (the higher the noise dose, the lower the vibration dose and vice versa).

To propose a method for the calculation of the $T_{Cexp}$ for noise and HAV, firstly the energy dose for noise and HAV physical factors, and the combined energy dose index are defined as (in the subsequent derivations all time units are in hours):

- Noise Energy Dose. The noise energy dose received by a worker is defined as:

(6) $$Dnoise=\frac{I_{rec}}{I_{std}}$$

where $I_{rec}$ stands for the average noise intensity (watts per square meters, W/m^2^) received by the worker in his/her working time $T_{e}$ and $I_{std}$ is the average noise intensity establishes by standards. In our case, based on Directive-2003/10/CE [75], this $I_{std}$ is set up as the average intensity equivalent to the criterium level (Upper EAV = 85 dBA) for a daily (8 h) exposure. If a worker has been exposed to a single exposition level $L_{Aeq,Ten}$ during a measurement time Ten (measurement time used to calculate the equivalent noise level $L_{Aeq,Ten}$), the received intensity in the time reference period (8 h) is equivalent to Equation (7).

(7) $$I_{rec}=10^{-12}\frac{1}{8}T_{e}10^{LAeq,Ten/10}$$

The Noise Energy Dose (Dnoise*) for* this case becomes:

(8) $$Dnoise=\frac{T_{e}}{8}10^{\frac{LAeq,Ten-85}{10}}$$

A value of 1 or 100% means that, in terms of energy, the worker has received the total amount of energy allowed by the chosen standard.

- HAV Energy Dose. The HAV energy dose is defined as the assessment of the exposure level to HAV is based on the calculation of daily exposure, which can be measured using the methods of ISO 5349-1:2001 and ISO 5349-2:2002 [72,73].
  (9) $$Dvibration=\frac{E_{rec}}{E_{std}}$$
  where $E_{rec}$ stands for the average HAV average energy measured during the use of a given machinery by the worker during his/her working usage time $T_{e}$ and $E_{std}$ is the HAV average energy derived from the standards. In our case, Directive 2002/44/EC [68] establishes the EAV for the daily (8 h) Acceleration Exposure *A*(8) as 2.5 m/s^2^ and this value is chosen as a criterion. Therefore, if a worker has been exposed to a single exposition level using a machine that generates HAV equivalent continuous acceleration over an $T_{ev}$ (h) period, calculated as a rms value, $a_{hv,eq}$, the HAV energy dose in the time reference period (8 h) is equivalent to Equation (10).

(10) $$Dvibration=\frac{a_{hveq}^{2}T_{ev}}{2.5^{2}\;8}$$

A value of 1 or 100% means that in terms of energy, the worker has received the total amount of energy allowed by the standard chosen.

- Combined Noise and HAV energy dose. This index is defined by a combination of the two indexes defined above. Since a value of any of the above indexes equal to 1 means that in terms of energy, the worker has received the total amount of energy allowed by the regulation. The combined noise and HAV energy (Dcombined) dose is defined consequently as the weighted arithmetic mean of both energy doses:
  (11) $$Dcombined=k\frac{w_{1}Dnoise+w_{2}Dvibration}{w_{1}+w_{2}}$$
  where $w_{1}$ and $w_{2}$ stand for the selected weighting factors for the noise and HAV energy dose respectively, and k is a normalizing factor. These factors can be selected by the prevention professionals as a value ranging from 0 to 1, depending on the relative relevance assigned to each physical factor by the prevention professionals, so the ratio $w_{1}$/$w_{2}$ means the relative relevance of one physical agent compared to the other one. The prevention professional, according to the worker health record, can adjust the weights so the relative importance of both agents can be taken into account at the discretion of the practitioner. In our case, we consider that both agents have the same relevance, i.e., the same relevance is given to noise and HAV energy exposure.

For this reason, both weighting factors are chosen as the same ones, and to maintain the maximum recommended energy exposure as the value of 1 (as in the case of individual doses), in this case the Dcombined is finally defined as the arithmetic sum of both doses.

(12) Dcombined=Dnoise+Dvibration 

Once the Dcombined index has been defined, the next step is to propose a method to obtain the $T_{Cexp}$. To this aim, if the worker is exposed both to noise (measured as the noise exposition level $L_{Aeq,Ten}$) and HAV (measured as the HAV equivalent continuous acceleration $a_{hv,eq}$ over the time $T_{ev}$), the following requirement should be fulfilled:

Criterion for action: Dcombined≤1
This criterion in fact also implies that Dvibration<1 and Dnoise<1

If this criterion is chosen, the occupational risk prevention professional should select exposure times for HAV and noise to assure the Dcombined is less than 1. These times will be denoted as $T_{CexpNoise}$ and $T_{CexpVib}$ and they will be connected. In this case, let us assume that one worker is exposed in their workplace to noise (characterised by the equivalent noise exposition level $L_{Aeq,Ten}$, measured over a prescribed time (Ten) and HAV (measured as the HAV equivalent continuous acceleration $a_{hv,eq}$ over a prescribed time $T_{ev}$). Our goal is to select the subsets of matching pairs ($T_{CexpNoise}$, $T_{CexpVib}$) that will keep the Dcombined index under 1, as the criteria we established.

The proposed method works as follows:

Step. 1. Given the noise exposition level of the worker $L_{Aeq,Ten}$, the maximum time allowed with this agent alone is calculated, i.e., the value corresponding to this pair ($T_{CexpNoise}$, 0). For this calculation Dnoise is equal to 1 and $T_{CexpNoise}$ is given by:

(13) $$T_{CexpNoiseMax}=8*10^{\frac{85-LAeq,Ten}{10}};\;\mathrm{for}\;\mathrm{the}\;\mathrm{case}\;T_{CexpVib}=0$$

Step. 2. The equivalent continuous acceleration $a_{hv,eq}$ over a prescribed time $T_{ev}$ is shown in Equation (1) and it is calculated as the maximum time allowed with this agent alone, i.e., the value corresponding to this pair (0, $T_{CexpVib}$). For this calculation Dvibration is equal to 1 and $T_{CexpVib}$ is given by:

(14) $$T_{CexpVibMax}=\frac{2.5^{2}*8}{a_{hveq}^{2}}\;;\;\mathrm{for}\;\mathrm{the}\;\mathrm{case}\;T_{CexpNoise}=0$$

Step. 3. Once the maximum numbers of the matching pairs have been established $\left(T_{CexpNoiseMax},\;T_{CexpVibMax}\right)$, the intermediate values of the time matching pairs are calculated. For this task, one can proceed as follows:

Step. 3.1. In the intervals of numbers ranging from 0 and $T_{CexpVibMax}$, there is fixed a set of values (namely N values) for which it is desired to calculate the matching pairs ($T_{CexpNoise}$,$T_{CexpVib}$). These values are denoted as $T_{CexpVib_{i}}$= $T_{CexpVibMax}$* i/N, i =1…N. For each one of the N values of $T_{CexpVib_{i}}$, the corresponding pair of $T_{CexpNoise_{i}}$ is calculated as follows:

- HAV Energy Doses $Dvibration_{i}$ are calculated for the $T_{CexpVib_{i}}$, i =1…N.
  (15) $$Dvibration_{i}=\frac{a_{hveq}^{2}\;T_{CexpVib_{i}}}{2.5^{2}*8}$$

Noise Energy Dose $Dnoise_{i}$ are calculated for i =1…N, maintaining the criterion that the Combined Noise and HAV energy dose must be 1 at the most.

(16) $$Dnoise_{i}=1-Dvibration_{i}\;$$

- Finally $T_{CexpNoise_{i}}$ is calculated, i.e., the maximum time given the calculated dose $Dnoise_{i}$, i.e., the value corresponding to this pair ($T_{CexpNoise_{i}}$,$T_{CexpVib_{i}}$). For this calculation, $T_{CexpNoise_{i}}$ is given by:
  (17) $$T_{CexpNoise_{i}}=8*Dnoise_{i}*10^{\frac{85-LAeq,Ten}{10}}$$

This procedure can be also replicated for the other case, i.e., when the fixed set of values comes from times of noise exposure. In this case, the procedure will be as follows:

Step. 3.2. In the intervals of numbers ranging from 0 and $T_{CexpNoiseMax}$, it is fixed a set of values (namely N values) for which it is desired to calculate the matching pairs ($T_{CexpNoise}$,$T_{CexpVib}$). These values are denoted as $T_{CexpNoise_{i}}$= $T_{CexpNoiseMax}$ * i/N, i =1…N. For each one of the N values of $T_{CexpNoise_{i}}$, the corresponding pair of $T_{CexpVib_{i}}$ is calculated as follows:

- Noise Energy Doses $Dnoise_{i}$ are calculated for the $T_{CexpNoise_{i}}$, i = 1…N.
  (18) $$Dnoise_{i}=\frac{T_{CexpNoise_{i}}}{8}10^{\frac{LAeq,Ten-85}{10}}$$

HAV Energy Dose $Dvibration_{i}$ are calculated for I = 1…N, keeping the criteria that the Combined Noise and Noise energy dose must be 1 at the most.

(19) $$Dvibration_{i}=1-Dnoise_{i}$$

- Finally, the it is calculated $T_{CexpVib_{i}}$, i.e., the maximum time given the calculated dose $Dvibration_{i}$, i.e., it is calculated the value corresponding to this pair ($T_{CexpNoise_{i}}$,$T_{CexpVib_{i}}$). For this calculation, $T_{CexpVib_{i}}$ is given by:
  (20) $$T_{CexpVib_{i}}=\frac{Dvibration_{i}*2.5^{2}*8}{a_{hveq}^{2}}$$

Finally for each specific case of a worker, the matching pairs ($T_{CexpNoise_{i}}$, $T_{CexpVib_{i}}$) are calculated that will keep the Dcombined index under 1. If the worker maintains below their exposure times in these pairs, it is assured that the combined dose will be kept less than 1, and so the energy received by both vibrations and noise is below the values that the standards consider as exposure values that give rise to an action. This is a safety measure that safety managers may adopt to protect the workers from adverse health effects.

In the case that the worker’s exposure originates from a given machine that generates both noise and vibrations, the procedure for the calculation of the recommended exposure time based on the combined index becomes as follows:

1. Let us suppose that a given worker uses a machine that generates noise and HAV exposure to the worker. This machine is characterized by the noise level $L_{Aeq,Ten}$ (noise equivalent level during a measurement time Ten) and HAV (measured as the HAV equivalent continuous acceleration $a_{hv,eq}$ over the measurement time $T_{ev}$).
2. The criterion is that the Combined Noise and HAV energy dose becomes lesser than 1, i.e.,
  (21) $$Dcombined=Dnoise+Dvibration=\frac{T_{Cexp}}{8}10^{\frac{LAeq,Ten-85}{10}}+\frac{a_{hveq}^{2}T_{Cexp}}{2.5^{2}*8}\leq 1$$

So, the recommended exposure time $T_{Cexp}$ can be obtained as:

(22) $$T_{Cexp}\leq \;\frac{8}{10^{\frac{LAeq,Ten-85}{10}}+\frac{a_{hveq}^{2}}{2.5^{2}}}$$

3. The maximum exposure time $T_{CexpMax}\;$ can be calculated from the equation above when the equality is accomplished:
  (23) $$T_{CexpMax}=\frac{8}{10^{\frac{LAeq,Ten-85}{10}}+\frac{a_{hveq}^{2}}{2.5^{2}}}$$

In this case the combined dose will be kept as 1, and so the energy received by both vibrations and noise is below the values that the standards consider as exposure values that give rise to an action. This can be a safety criterium that preventionists may adopt to protect the workers from adverse health effects in case of combined exposure.

## 5. Application Example: Study Case in Workers in the Olive Sector 

The proposed index can be applied to any activity where workers are exposed to both noise and HAV simultaneously. As an example of its use the activity of olive harvesting by hand-guided machines was selected in this research. 

This study was carried out in Andalusia, a region located in the south of Spain, during the harvesting period from November to March. As mentioned above, the number of working days are accounted for as about 19 millions [85] in an average production season.

### 5.1. Data Acquisition and Measurement 

**Figure 5:**
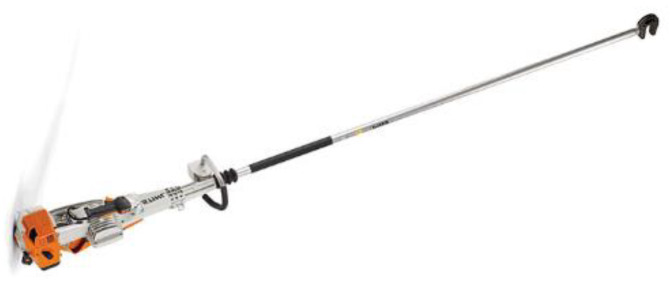
Hook type olive harvesters.

In the olive sector there are a wide variety of hand-held machines that achieve fruit detachment. In Andalusia, the most common used are the hook type olive harvesters, due to their productivity and the minimum damage to the tree shoots (see Figure 5).

The hook type olive harvesters consist of a one-piece rod of variable length ranging from 1.5 to 3 m with an inverted v-shaped end covered with shock-absorbing material. The operating system differs from one brand to another, and most of them are petrol-powered, with a two-stroke combustion engine of between 1 and 3.5 HP. The characteristics of the measured machine are shown in Table 2.

**Figure 6:**
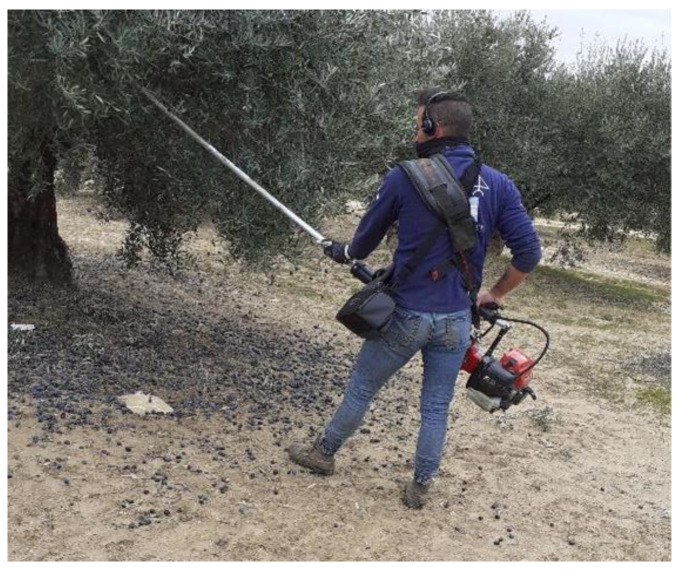
Worker equipped with hook type olive harvesters and measurement sensors.

During the measurements, the worker was provided with the sensors and equipment described in Section 3.2 Equipment Used. Figure 6 shows where the sensors were placed: the vibration sensor in the palm of the hand and the noise sensor over the ears to register the worker exposition. The data logger is placed in a bag to allow the operator to perform his usual work operations. The vibration values of harvesters were measured and analysed for both right and left hands and the noise pressure level was measured at ear place of the operator.

**Figure 7:**
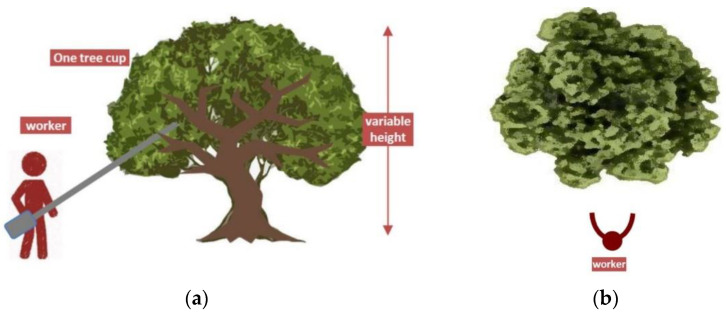
(**a**) Diagram of the worker’s position in the lateral view; (**b**) diagram of the worker’s position in the front view.

The measurements were carried out under real working conditions during a normal working day in several plantations, all located in the region of Andalusia. The type of plantation being studied is that of traditional olive tree plantations, with one or several trunks, but with a single crown; the height of the top is variable, ranging from 3 to 5 metres, and the worker moves around the tree (see Figure 7). The worker holds the tool with both hands and places the hook on the medium-thick tree branch, the operation shortens and lengthens the pole producing vibration on the tree to release the fruit (olives). In this operation, the tool transmits HAV to the worker in both hands, and it emits noise.

The workers participating in this study were selected because they are qualified workers with a large experience in the operation of selected machinery, according to the characteristics included in the ISO 5349-1:2001 and ISO 5349-2:2002 [72,73]. The measurement was carried out during their working day. The only criteria to select the workers were those based on the experience regardless of their physical condition, sex or age. In this case all the workers were male and aged between 30 and 60 years.

### 5.2. Measuring Results and Exposure Time Calculation

First of all, the process of characterization of the hook type olive harvesters in terms of noise emission and HAV was performed. The measurements were taken according to the ISO 5349-1:2001 and ISO 5349-2:2002 for HAV [72,73] and ISO 1999:2013 [101] for noise assessment. The results obtained from the measurement of the model 1: hook type olive harvesters are shown in Table 3.

According to Equation (2) the obtained value of $a_{hv}$ is 6.33 m/s^2^. Likewise, the value of $L_{Aeq,T}\;$is 97.7 dB(A).

Once the hook type olive harvesters are characterized, two types of calculations are performed. Firstly, the maximum exposure time for each one of the physical agents is calculated independently. The criterion used was that of the previous Section 4, by keeping the energy dose for noise and HAV physical agents up to 1 independently. Therefore, $T_{exp}$ for HAV was calculated in order not to exceed the EAV value of *A*(8) according to Table 1 (i.e., 2.5 m/s^2^) and in accordance with Equation (14). The same procedure was applied to the calculation of $T_{exp}$ for noise. These calcultated exposure times coincide with the so-called $T_{CexpVibMax}$ and $T_{CexpNoiseMax}$ according to Section 4 (see Table 4).

In this case, the recommended exposures times will be 0.421 h if noise is considered and 1248 h if HAV is considered. However, in this case the worker is exposed to both noise and HAV since the worker’s exposure originates from a given machine that generates both noise and vibrations. In this case, the suggested procedure take into account both exposures and the recommended maximum exposition time $T_{CexpMax}$ shall be calculated according to Equation (23), the result being 0.315 h.

As can be expected, if we take into account both exposures, the recommended combined exposure time $T_{CexpMax}\;$ is less than $T_{CexpVibMax}$ or $T_{CexpNoiseMax}$. Table 5 lists the values of the Dvibration and Dnoise for this combined recommended exposure time $T_{CexpMax}$, by ensuring that the sum of both noise and HAV doses becomes equal to Equation (21).

The value of *A*(8) according to Equation (1) is 1.3 m/s^2^ and the value of $L_{Aeq,d}\;$ is 83.7 dBA. As can be seen, both are below the EAV values listed in Table 1.

## 6. Discussion and Limitations of the Suggested Approach

Simultaneous exposure to HAV and noise is common in different industrial and construction or production sectors. There are two possible situations where the worker becomes exposure to both physical agents:

(1) There are two different sources that may affect the worker; one being workers who are exposed to HAV through the normal operation of machinery and the other being another source not in contact that emits noise whose wave propagation reaches the worker. Since there is simultaneous exposure to both noise and HAV, the activity implies the worker receives exposure to both agents and it should be considered preventively.
(2) The second general possibility is that there is one single source or machine, which transmits both agents simultaneously to the worker.

Of course, both situations may also appear in some specific situations.

The proposal in this research seeks to propose a recommended time exposure in both cases considered above. In the first case, when the worker is exposed to two or more sources, but the activity implies that they operate simultaneously, the maximum exposure time for noise and HAV can be calculated for both sources, so as not to exceed the maximum dose values. In this case two maximum recommended times are obtained for the two agents, so the safety manager should choose the lower of the two to maintain the combined dose as less than one. Another situation in this case can also arise if during a working day the worker receives HAV from one machine and noise from another source, but the activity does not involve simultaneous operation. The safety manager will allow each machine to be used for the maximum time calculated for an 8-h working day and which does not exceed the EAV values shown in Table 4. 

In the second case, the noise and HAV comes from the same source as is usual in most sectors the machinery that transmits HAV also emits noise, so the worker receives both energies at the same time. The suggested procedure calculated a recommended exposure time using this machine to avoid the combined dose to be exceeded. This case was the study case shown in the preceding section where the recommended time was 0.315 h, less than the 0.421 h if only noise is considered or 1248 h if only HAV is considered. 

It should be pointed out that this research was made considering real data in the olive sector, where the workers’ exposure comes from hook type olive harvesters such as those considered in the preceding section. Nevertheless, it can be considered for other cases where the reduction in the recommended exposure time becomes greater.

Concerning the underlying assumptions of the proposed procedure, Hypotheses 1 and 2 summarise the main results on which the proposed approach is based. The main assumption is that the total effect caused by a combination of noise and vibration can be determined from a summation of the energy received by the worker coming from noise and vibration sources. This does not deny the possibility of synergistic effects on combined health effects, but it assumes that the sum of energies from noise and vibration sources limits the exposure time in an additive way. This approach may be logical, unless further information related on that the effects are synergistic or complex interaction model are available. At the current stage of knowledge, this combined effect could be considered as additive as an initial approach. This approach is similar, for example, to that assumed with chemical agents in those cases where there is not established a complex interaction model.

In this regard, the literature on health effects on combined exposure to both noise and vibration is not conclusive. Most of the research establishes the appearance of combined effects, and some of them indicates the possibility of synergistic effects but without a clear underlying model or pattern. For this research, we adopt the main ideas suggested in the literature review, i.e.,

(a) There is a relationship between noise and HAV, and so there are combined effects that demand efforts aimed at reducing and managing occupational noise and vibration exposures. The current occupational and environmental noise regulations may significantly underestimate the adverse health and societal cost of noise-induced hearing loss Shepard et al. [34], and so other approaches as suggested in this proposal may be useful.

(b) The results indicate that there are interactions between noise and vibration, but the effect may not be simple or consistent in some studies. A reasonable approximation to the total annoyance caused by a combination of noise and vibration can be determined from a summation of the effects of the individual agents Howart H, Griffin M. [105]. This is the approach followed in this research.

The hypothesis that the energy levels are summative represents a valuable measure of combined exposure while no other specific information is available or tested. Nevertheless, if synergistic effects are considered or more complex interaction models are proven or needed in some cases, the proposed additive model on energies may be no longer valid and it may underestimate effects of combined exposure. This fact results in a limitation of the proposed model and for those cases where these synergistic effects are clear, this procedure should be applied with caution, and its results should be considered as a first guide to reduce exposure. In this case, the suggested procedure should be revised introducing a new index definition based on the more complex interaction models from the synergistic effects.

Therefore, this suggested index and this proposal should be applied with caution in those cases where it is suspected the appearance of complex or synergistic interactions between noise and vibration. In this case, an approach taking into account not only energy of signals but also some other parameters could be a good starting point to tackle the problem of specific health effects or more complex interactions. For example, differences in exposure characteristics of the physical signals in terms of peak levels, number of peaks, frequency of peak levels, noise and HAV spectrum etc., specific energy levels for a certain noise exposure or a certain HAV level as occupational stressors could be some interesting parameters to deal with in these cases and a future field or research.

In summary, this paper has suggested a procedure for the calculation of a recommended maximum exposure time, taking into account the amount of energy received by the workers. The effects of noise and HAV on the workers’ health depend on the energy dose received by the workers. The Directive-2003/10/EC [75] established the Upper EAV for the daily (8 h) Equivalent Continuous Noise Pressure Level in dB(A)) and Directive 2002/44/EC [68] establishes the EAV for the daily (8 h) Acceleration Exposure *A*(8). These limits are defined for the analysis of individual agents based on the wave and vibration energy received by the workers. The assumption of the simultaneous exposure should be considered, and as the energy dose is coming from both agents, the suggested procedure proposes a recommendation to limit the time exposure of the worker when both agents are present, to be sure that the values for an action to be taken are not exceeded for both agents. The proposal should be considered as a recommendation based on the amount of total energy received with reference to the total dose for both agents, and it does not consider possible synergetic effects or combined health issues arising for combined expositions, since in this case most tighter recommendations should be taken into account.

Finally, although equal relevance has been established in the suggested procedure in Equation (11) for the two physical agents, the safety manager could decide to apply different relevance to one or other of the agents, based on the data or medical record of the worker. For example, in cases of previous hearing impairment or hand-arm vascular problems, different values for the weighting w_1_ or w_2_ in Equation (11) could be selected for the process of calculating the combined dose. Thus, the exposure times can be calculated taking into account this different relevance of both physical agents using the same procedure described in the application example. It should be noted that this research does not seek to define or establish any new criteria levels or standards, but only a combined use of energy doses from these standards is suggested as a tool to establish recommendations for exposure in workers. As it was commented in the introduction, this approach is similar to other well-established procedures in the scientific literature based on the current knowledge on the combined health effects. It provides a method to calculate exposure times beyond other hands-on criteria or broad guides based on practice rather than theory. In future research it will be studied how the weighting factors $w_{1}\;$or $w_{2}$ in Equation (11) can be selected to take into account particular variables such as the specific tasks performed by the worker, previous pathologies and susceptibility of the worker to any of these physical agents. In this future approach, the safety manager expertise will be embedded in a procedure based on multicriteria decision-making techniques.

## 7. Conclusions

This paper suggests a combined energy dose index to be taken as a basis for the calculation of a recommended maximum exposure time based on the criterion of not exceeding the Exposure Action Value for noise and HAV. Based on this combined index, several situations can arise, and the suggested procedure allows the maximum recommended times for noise or HAV in the case of multiple sources operating simultaneously or not, and the maximum recommended exposure time in the case of one source simultaneously generating HAV and noise to be calculated.

The application of this index in either of the two situations would always offer exposure times below the exposure times calculated if both agents are considered independently. This procedure should be considered as an aid to the safety manager to ensure the health of workers and as a precautionary measure for workers with simultaneous exposure to both agents.

Future research will be devoted to further characterise and analyse the use of this methodology based on this energy dose combined index in workers not only in the olive sector, but also from other industrial sectors which operate with different machinery generating both noise and HAV. In addition, how to provide a way to integrate specific health issues of workers in the combined index will be explored.

## Figures and Tables

**Table 1:** The daily limit values EAV and ELV defined by Directive 2002/44/EC [68] and Directive 2003/10/EC [75].

Directive 2002/44/EC		Directive 2003/10/EC		
Daily Limit Values	*A*(8)	Daily Limit Values	$L_{Aeq,d}$ [dBA]	$L_{Ppeak}$ [dBC]
EAV	2.5 m/s^2^	Upper EAV	85	137
ELV	5 m/s^2^	ELV	87	140

**Table 2:** Measured machine. Noise and vibration values declared by the manufacturer.

Model	Engine cc	Power	Noise ^1^	Vibration ^2^
1	48.7 cm^3^	(3.0 HP) (2.2 KW)	$L_{eq}$ = 102 dB(A) $L_{w}$ = 113 dB(A)	$a_{hv,eq}\;$left = 5.7 m/s^2^ $a_{hv,eq}$ right = 5.7 m/s^2^

^1^ ISO 11201:2010 [102] Noise emitted by machinery and equipment. ^2^ ISO-20643:2008 [100] Mechanical vibration. Hand-held and hand-guided machinery.

**Table 3:** Noise and vibration values measured in simultaneous time periods (for each operator).

Operator	X m/s^2^	Y m/s^2^	Z m/s^2^	Vibration $a_{hv}$	Arithmetic Average	Noise $L_{Aeq,T}$	Logarithmic Average
1	2.707	5.609	2.553	6.731		97.3	
	3.105	3.508	2.506	5.312		93.8	
	4.069	3.926	2.239	6.081		94.2	
	6.714	5.176	3.010	8.996		96.7	
	2.698	2.767	2.104	4.400		90.6	
					6.304		95.1
2	2.917	3.999	1.409	5.146		96.4	
	2.301	3.720	2.352	4.966		97.7	
	1.455	2.633	0.985	3.165		82.6	
	1.834	1.869	1.070	2.828		94.2	
	5.135	12.488	3.560	13.964		97.1	
					6.014		95.6
3	4.074	9.057	4.534	10.917		99.3	
	5.495	6.516	2.884	8.998		100.1	
	1.288	1.991	1.660	2.894		100.1	
	2.415	3.681	2.941	5.294		97.6	
	2.701	3.627	2.645	5.238		103.2	
					6.668		100.4

**Table 4:** Maximum exposure times for noise and vibration taken independently.

Noise (Equation (13))	HAV (Equation (14))
$T_{CexpNoiseMax}=0.421\;h$	$T_{CexpVibMax}=$1.248 h

**Table 5:** Dvibration and Dnoise achieved for the combined recommended maximum exposure time $T_{CexpMax}$.

Dnoise (Equation (8))	Dvibration (Equation (10))
0.748	0.252

## Data Availability

Data are provided upon request to the corresponding author.
